# Effect of Whey Protein Supplementation on Physical Performance and Body Composition in Army Initial Entry Training Soldiers

**DOI:** 10.3390/nu10091248

**Published:** 2018-09-06

**Authors:** Jeremy S. McAdam, Kaitlin D. McGinnis, Darren T. Beck, Cody T. Haun, Matthew A. Romero, Petey W. Mumford, Paul A. Roberson, Kaelin C. Young, Keith R. Lohse, Christopher M. Lockwood, Michael D. Roberts, JoEllen M. Sefton

**Affiliations:** 1 Warrior Research Center, School of Kinesiology, Auburn University, Auburn, AL 36849, USA; jsm0039@auburn.edu (J.S.M.); kdm0031@auburn.edu (K.D.M.); 2 Molecular and Applied Sciences Laboratory, School of Kinesiology, Auburn University, Auburn, AL 36849, USA; dbeck@auburn.vcom.edu (D.T.B.); cth0023@auburn.edu (C.T.H.); mzr0049@auburn.edu (M.A.R.); pwm0009@auburn.edu (P.W.M.); par0021@auburn.edu (P.A.R.); kyoung@auburn.vcom.edu (K.C.Y.); mdr0024@auburn.edu (M.D.R.); 3 Department of Cell Biology and Physiology, Edward Via College of Osteopathic Medicine (Auburn Campus), Auburn, AL 36849, USA; 4 Neurorehabilitation Informatics Lab, Department of Health, Kinesiology, & Recreation, University of Utah, Salt Lake City, UT 84112, USA; Keith.Lohse@health.utah.edu; 5 Lockwood LLC, Draper, UT 84020, USA; chris@drchrislockwood.com

**Keywords:** soldiers, military, nutrition, recovery, basic training

## Abstract

We investigated the effects of whey protein (WP) supplementation on body composition and physical performance in soldiers participating in Army Initial Entry Training (IET). Sixty-nine, male United States Army soldiers volunteered for supplementation with either twice daily whey protein (WP, 77 g/day protein, ~580 kcal/day; *n* = 34, age = 19 ± 1 year, height = 173 ± 6 cm, weight = 73.4 ± 12.7 kg) or energy-matched carbohydrate (CHO) drinks (CHO, 127 g/day carbohydrate, ~580 kcal/day; *n* = 35, age = 19 ± 1 year, height = 173 ± 5 cm, weight = 72.3 ± 10.9 kg) for eight weeks during IET. Physical performance was evaluated using the Army Physical Fitness Test during weeks two and eight. Body composition was assessed using 7-site skinfold assessment during weeks one and nine. Post-testing push-up performance averaged 7 repetitions higher in the WP compared to the CHO group (F = 10.1, *p* < 0.001) when controlling for baseline. There was a significant decrease in fat mass at post-training (F = 4.63, *p* = 0.04), but no significant change in run performance (F = 3.50, *p* = 0.065) or fat-free mass (F = 0.70, *p* = 0.41). Effect sizes for fat-free mass gains were large for both the WP (Cohen’s d = 0.44) and CHO (Cohen’s d = 0.42) groups. WP had a large effect on fat mass (FM) loss (Cohen’s d = −0.67), while CHO had a medium effect (Cohen’s d = −0.40). Twice daily supplementation with WP improved push-up performance and potentiated reductions in fat mass during IET training in comparison to CHO supplementation.

## 1. Introduction

Army initial entry training (IET) is a physically demanding program consisting of large volumes of strength and endurance activities [1]. IET is the first step in a soldier’s military career, and the goal of IET is to transform civilians into soldiers who have the strength and endurance needed to meet the demands of deployment and remain healthy throughout a rigorous military career. An increasing percentage of soldiers report to IET with low levels of physical fitness, which is a primary contributor to the high rates of musculoskeletal injuries found in the military population [2]. To help combat this problem the Army has implemented strategies such as the Army Performance Triad which focuses on improving sleep, exercise, and nutrition [3]. The Army has also changed approaches to physical training during IET [4,5], and is currently piloting extending length of IET to improve proficiency, health, and performance. 

Nutritional intake is a key component of the physiological adaptation to training [6]. Inadequate nutritional intake can result in a negative energy balance, which can adversely impact exercise performance and adaptation [7]. Investigations quantifying training have revealed that IET soldiers spend approximately 6–7 h per day performing light to very vigorous physical activity [8,9]. Dietary intake of IET soldiers has been reported to be between 1900 [10] and 2600 [9] calories per day, which may be inadequate to meet the demands of training [9]. Notably, the latter study reported IET soldiers on average consumed 540 kcals less than the estimated energy expenditure per day during IET [9]. Optimal protein intake for individuals participating in large volumes of training has been reported to be 1.5 to 2.0 g/kg of bodyweight per day [11,12]. Importantly, energy restriction like that noted during IET may increase these needs [13]. Previous research [13] and organizational recommendations [6] suggest protein intakes between 2–3 g/kg may be necessary during periods of energy restriction. Collectively these dietary and training volume reports suggest soldiers in IET may be functioning in a negative energy balance and may need to increase protein intake to support adaptation to training [8,14].

Protein supplementation may be a strategy to help combat nutritional deficits during military training [14]. However, only one study to date has examined the use of protein supplementation in United States soldiers engaging in IET [15]. In this particular study, marine recruits were supplemented with 10 g of casein (protein source derived from milk) once daily throughout 54 days of basic training. All participants experienced losses in lean mass. However, the casein-supplemented soldiers experienced a 33% reduction in total medical visits and a 37% reduction in medical visits due to musculoskeletal injury [15]. Mixed-macronutrient supplementation has also been employed to help optimize nutritional intake during non-IET military training. The administration of a mixed-macronutrient supplement (45% carbohydrate, 40% fat, and 15% protein) to soldiers undergoing section commander’s battle course in the United Kingdom resulted in the attenuation of muscle mass loss and physical performance decrements, whereas un-supplemented soldiers experienced greater decrements in these measures [16].

Whey protein (WP) is considered a higher quality protein supplement in comparison to other supplemental protein sources [17]. WP is rapidly digested, contains a high content of branched chain and essential amino acids, and possesses bioactive peptides that are exclusive from other protein sources [18,19]. Research suggests these characteristics result in an improved ability to stimulate muscle protein synthesis thereby improving net protein balance and improving recovery through skeletal muscle adaptation and repair [19]. WP supplementation has been reported to improve strength [20,21], increase lean mass [20,21], and facilitate recovery of force production between repeated bouts of exercise in resistance-trained individuals [22]. WP supplementation has also been reported to improve run time, cycle time to exhaustion, and reduce serum creatine kinase levels, suggesting WP facilitates recovery in subjects engaged in high-volume endurance training [23,24]. 

Military training contains a unique combination of strength and endurance-related activities in the form of structured physical training, as well as in required military (functional) tasks such as ruck marches and land navigation [1]. Therefore, increasing the intake of high-quality protein during IET through WP supplementation may assist in recovery and adaptation while also providing additional calories to help match the energy demands of training. The purpose of this study was to examine the effects of WP supplementation in comparison to a carbohydrate (CHO) placebo on physical performance and body composition during Army IET. We hypothesized that WP supplementation would result in greater improvements in body composition, specifically increase fat-free mass, and also improve physical performance compared to an energy-matched CHO control group.

## 2. Methods

### 2.1. Study Design

This study employed a repeated measures, double-blind, parallel groups study design. The Auburn University Institutional Review Board, Army Institutional Review Board, and the Director, Research & Analysis Directorate Army Center approved the study. Participants were apparently healthy IET males at least 18 years of age, with no apparent disease or musculoskeletal injury, no allergy to milk or WP, and not supplementing or have supplemented with WP or any other ergogenic aid within the past three months. Each potential participant received verbal explanation of the study, and written consent was obtained from those wishing to participate.

**Figure 1:**
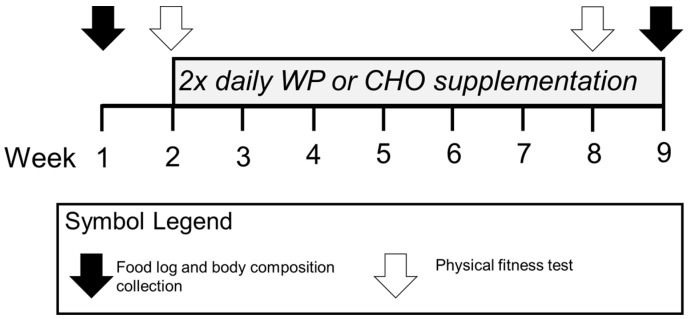
Summary of measures collected during Initial Entry Training (IET). This figure illustrates the logistics of the 8-week supplement intervention.

A total of 69 healthy male subjects from one training unit at Fort Benning Georgia, consented to participate and completed the study. Figure 1 illustrates the timeline of measurements.

IET soldiers from two platoons received WP, and two platoons of the same training unit received a taste and calorie-matched CHO placebo supplement. Two servings per day were administered by drill sergeants; one after morning physical fitness training, and the second serving before bedtime. Supplements were provided in powder form in coded, single serving packets. All supplements were manufactured at JW Nutritional, LLC (Allen, TX, USA), a United States Food and Drug Administration cGMP-compliant facility independently audited and pre-qualified by Obvium*Q, LLC (Phoenix, AZ, USA), a GMP regulatory compliance firm. Personnel at JW Nutritional, LLC and C.M.L. (Lockwood, LLC; Draper, UT, USA) formulated supplements to match for taste. These entities also maintained blinding of groups, and each supplement was assigned a randomly generated item number. The research team and participants were blinded to the contents of the packets until data collection was completed. Manufacturing batch records for production of each of the supplements were reviewed by a trained, independent expert in dietary supplement quality control, taste, and assurance (C.M.L.) before approval for use within the present study. The nutritional profile and amino acid content of both supplements were third-party tested by Covance Laboratories, Inc. (Madison, WI, USA) to verify identity, purity, potency, and composition of the packets. One WP serving provided 293 total kcals; consisting of 38.6 g of protein (Power Crunch^®^ ProtoWhey^®^ (BioNutritional Research Group; Irvine, CA, USA) as agglomerated, partially hydrolyzed (12.5% degree of hydrolysis) 80% whey protein concentrate (Hilmar^®^ 8360; Hilmar Ingredients, Hilmar, CA USA)), 19.0 g carbohydrates, 7.5 g fat, and 20.1 g and 9.5 g of essential and branched chain amino acids. One CHO serving provided 291 total kcals, 0.5 g protein, 63.4 g carbohydrates, 3.9 g of fat, and 0.1 g and 0.0 g essential and branched chain amino acids. A member of the research team delivered and checked the supplement supply weekly to assess for compliance. Participants also self-reported any missed servings and a researcher asked each participant if any servings had been missed at each assessment. Additionally, as is standard in IET, soldiers: (1) were required to live in barracks, and were under continual supervision of drill sergeants; (2) performed daily physical fitness and military training tightly regulated by unit leadership, scheduled from the time the soldiers wakes up until time for bed; (3) were not allowed to smoke or consume alcohol; (4) consumed all meals in the dining facility or at unit provided meals in the field, and; (5) no food is allowed in the barracks, and no beverages other than water are allowed outside of the scheduled meal times at the IET dining facility.

### 2.2. Dietary Analysis

Dietary intake was evaluated using diet recalls collected immediately following each meal on three days (Tuesday, Thursday, and Saturday) during weeks one and nine of IET. A more detailed methodology has been described in our previous work [9]. Briefly, participants were provided a diet log immediately after the meal that was pre-filled with food offerings was obtained from the battalion dining facility. Participants were asked to circle the food or drink item and amount consumed. Nutritional information for food items was collected from the Army Joint Culinary Center of Excellence (JCOE) website [25]. Information for food items not found on the JCOE menu were retrieved from the United States Department of Agriculture nutrition database [26]. Dietary intake was calculated by multiplying the portion of food consumed by the nutritional value of that food using R statistical software [27] for each meal and summed across each day. Participants who did not complete more than two days of diet logs were excluded from the dietary intake analyses. A full day was determined as completing a diet log for each of the three meals for that day. Overall, 27 in the WP group and 28 in the CHO group were included in the dietary analysis.

### 2.3. Body Composition and Performance Measures

Anthropometric measures were conducted following an overnight fast, prior to morning physical training and breakfast on the third day of the IET training cycle (pre-intervention) and at the same time of day during week nine (post-intervention) of IET training. First, urine specific gravity (USG) was evaluated the morning of testing using a handheld refractometer (Manual, Atago, Tokyo, Japan) to ensure participants were properly hydrated (USG below 1.030). Those with USG values above 1.030 were provided with water and re-tested to assure proper hydration before proceeding to the remaining assessments.

#### 2.3.1. Height and Weight

Height and weight were recorded using a Health-O-Meter professional scale (Model 500KL, Sunbeam products INC. Boca Raton, FL, USA) with IET soldiers wearing only Army issued physical training shorts, socks and underwear. Weights were collected to the nearest 0.1 kg.

#### 2.3.2. Body Composition

Skinfolds were measured by three trained technicians using skinfold calipers (Fabrication Enterprises, PO Box 1500 White Plains, NY, USA) as per the American College of Sports Medicine (ACSM) protocol for 7-site skinfold measures (chest, mid-axilla, abdomen, suprailiac, subscapular, triceps, and thigh) [28]. Duplicate measures from the right side of the body were taken in sequential order and averaged. A triplicate measure was assessed on a given site if the first two readings differed by ± 2.0 mm. The ACSM 7-site body density calculation was used and body composition calculated as follows:

(1) Body Fat Percent=((457/Body Density) − 414.2)/100

(2) Fat Mass (kg)=Body Fat Percent×Body Mass

(3) Fat Free Mass (kg)=Body Mass−Fat Mass 

Based upon within day test-retest measurements performed on 10 male volunteers, the intraclass correlation for the 7-site sum of skinfolds was 0.99, 1.00, and 0.99 for each of the three technicians that performed all skinfold measurements in the current study. Additionally, the measurements exhibited a high degree of correlation (*r* = 0.91) between all three technicians.

#### 2.3.3. Performance Assessments

The Army Physical Fitness Test (APFT) was performed during weeks two and eight of the intervention [4]. The APFT consisted of a 2-min push-up test, 2-min sit-up test, and 2-mile run test. The assessment process was supervised by experienced drill sergeants and cadre specifically trained to administer the APFT. Results were then supplied to the research team. A more detailed description of the APFT is previously described [4,29].

### 2.4. Statistical Analysis

Shapiro Wilk’s and Kolmogorov Smirnov tests were used to test the assumption that residuals were normally distributed across all levels of each dependent variable. Additionally, QQ plots were used to visualize residuals for each level of the variables. Square root transformations were performed and used in the analysis for dependent variables in which normality was violated for more than 75% of the measures. Maulchy’s test of sphericity was used to test the assumption of equality of variances. Greenhouse-Geisser corrections were used if sphericity was violated. Levene’s Test was used to evaluate the assumption of homogeneity. Statistical analyses were completed using R statistical software [27] and R Studio [30]. R programming packages included dplyr, tidyr, reshape2 ez, car, vars, ggplot2. An *a priori* alpha level of 0.05 was used as the criteria for determination of significant effects.

Dietary intake data was evaluated using a two-way (training × supplement group) mixed factorial analysis of variance (ANOVA). If group by time interactions were detected, paired samples t-tests were used to evaluate simple main effects of time, and independent samples t-test were used to evaluate group differences at pre and post-intervention.

Physical performance (push-ups, sit-ups, and run time) and body composition (body weight, FFM, FM) measures were evaluated using analysis of covariance (ANCOVA). Mean-centered baseline values for each variable were used as the covariate in the model, which reduces the error rate in ANCOVA models containing within-subjects factors [31]. ANCOVA improves the sensitivity of detecting group effects [31], which was our primary research interest. We also conducted a *post hoc* correlation test to determine whether initial FFM was associated with the change in FFM across training.

Cohen’s d effect sizes were calculated for each variable across training (pre- vs. post-intervention). Effect sizes were classified as small (d < 0.2), medium (d > 0.21, d < 0.5), or large (d > 0.51), and the results are presented as Cohen’s d effect size estimate.

Effect Size = mean(post) - mean (pre)/pooled standard deviation(4)

Pooled standard deviation = Square root ((SD (pre)^2^ + SD (post)^2^)/2) (5)

## 3. Results

A total of 69 IET soldiers participated in the study (WP: *n* = 34, age = 19 ± 1 year, height = 173 ± 6 cm, weight = 73.4 ± 12.7 kg; CHO: *n* = 35, age = 19 ± 1 year, height = 173 ± 5 cm, weight = 72.3 ± 10.9 kg). With the exception of FM, all other dependent variables did not violate the assumption of normality of residuals. Statistical differences were found for all time points and groups for FM except for post-intervention in the CHO group (WP: Pre-W = 0.93 *p* = 0.03, Post-W = 0.92, *p* = 0.01; CHO: Pre-W = 0.93, *p* = 0.04, Post-W = 0.94, *p* = 0.06). FM was square root transformed, normality of residuals was re-tested, and the data was normally distributed following transformation.

### 3.1. Dietary Intake

First, we analyzed dietary intake on only what was consumed at the dining facility at post-intervention (Post-NS) to determine if WP or CHO supplementation decreased food intake. There were no significant differences across the intervention or between groups for calorie or macronutrient intakes. We then compared dietary intake with the supplement nutrition added to total dietary intake at post-intervention (Post-SI). Calorie (F = 80.44, *p* < 0.001) and fat intake (F = 18.50, *p* < 0.001) increased across the intervention. There were significant group by time interactions for protein (F = 95.97, *p* < 0.001) and carbohydrate intake (F = 14.68, *p* < 0.001). Follow-up t-tests revealed the WP group increased protein (*t* = 14.28, *p* < 0.001) and carbohydrate (*t* = 3.71, *p* < 0.001) intake across IET; however, the CHO group only increased carbohydrate intake (*t* = 8.91, *p* < 0.001).

These results indicate soldiers in both the WP and CHO groups increased calorie, carbohydrate, and fat intake across IET, whereas only the WP group increased protein intake across IET. Dietary intake is summarized in Table 1 and significance is denoted accordingly.

### 3.2. Body Composition

After controlling for initial body weight (week one) there was no statistically significant difference between groups in body mass at post-intervention (week nine) (F = 0.93, *p* = 0.34), or initial weight by group interaction (2.05, *p* = 0.16). 

There was a significant difference in FM post-intervention after controlling for initial FM (F = 4.63, *p* = 0.04). There was no significant initial FM by group interaction (F = 1.30, *p* =0.26).

There was no statistically significant difference in FFM between the groups post-intervention when controlling for initial FFM (F = 0.70, *p* = 0.41). There was no initial FFM by group interaction (F = 1.90, *p* = 0.17). Post hoc testing revealed a significant inverse correlation between change in FFM across training with initial FFM (*r* = −0.45, *p* < 0.001), indicating that individuals with lower initial FFM were likely to have larger gains in FFM during IET. Body composition data is summarized in Table 2 and effect sizes for body composition and performance are summarized in Table 3. 

**Figure 2:**
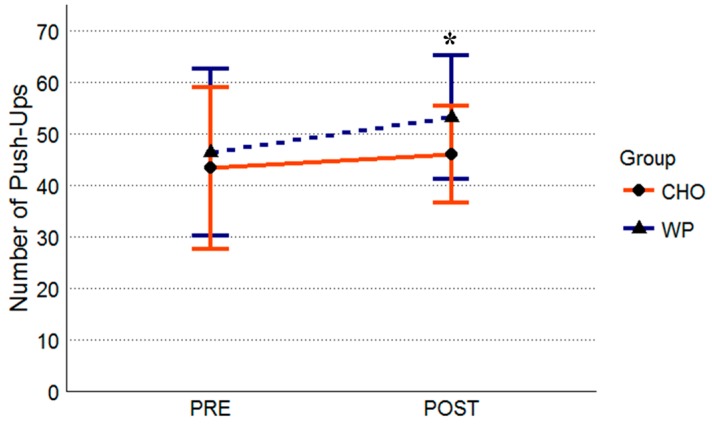
Push-up Performance. All data are presented as means ± standard deviation values. Abbreviations: WP, whey protein group (*n* = 34); CHO, carbohydrate-placebo group (*n* = 35). Symbols: *, indicates WP > CHO at POST.

After controlling for initial push-up performance there was a significant group difference at post-intervention (F = 10.02, *p* = 0.002). There was no significant initial push-up by group interaction (F = 0.25, *p* = 0.62). There was a difference of 6.87 push-ups on average between groups at post-intervention, with the WP performing more push-ups (53 ± 12) compared to the CHO (46 ± 9 push-ups) group at post-intervention (Figure 2). Effect sizes and mean differences for push-up performance are summarized in Table 3.

**Figure 3:**
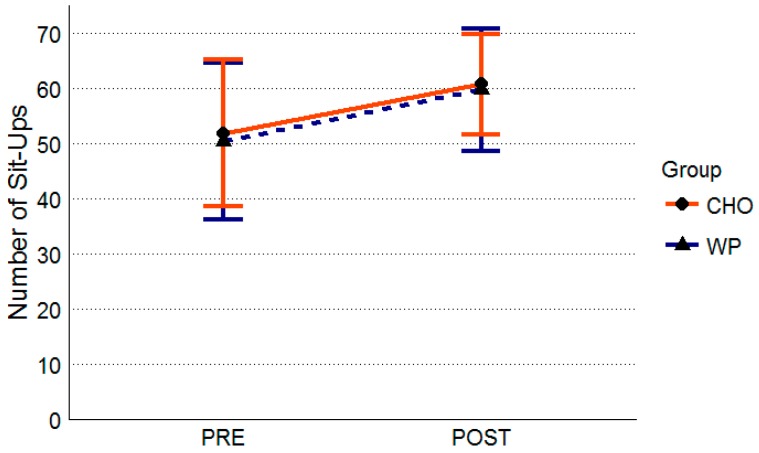
Sit-up Performance. All data are presented as means ± standard deviation values. Abbreviations: WP, whey protein group (*n* = 34); CHO, carbohydrate-placebo group (*n* = 35).

After controlling for initial sit-up performance there was no significant group difference at post-intervention (F = 0.02, *p* = 0.90). There was no significant initial sit-up by group interaction (F = 0.42, *p* = 0.52). WP group performed 50.4 ± 14.3 sit-ups pre- and 59.7 ± 11.0 post-intervention (Figure 3). The CHO group performed 51.8 ± 13.3 sit-ups pre and 60.7 ± 9.2 sit-ups post-intervention (Figure 3). Effect sizes and mean differences for sit-up performance are summarized in Table 3.

**Figure 4:**
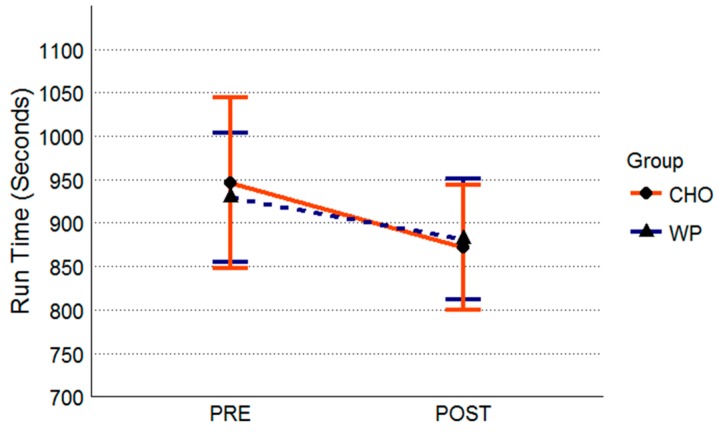
Run Performance. All data are presented as means ± standard deviation values. Abbreviations: WP, whey protein group (*n* = 34); CHO, carbohydrate-placebo group (*n* = 35).

After controlling for initial run performance there was no significant group difference at post-intervention (F = 3.64, *p* = 0.06). There was no significant initial run by group interaction (F = 2.89, *p* = 0.09). Post-intervention run performance was 14:41 (minutes:seconds) ± 1:9 in the WP group and 14:32 (minutes:seconds) ± 1:11 in the CHO group (Figure 4). Effect sizes and mean differences for run performance are summarized in Table 3.

## 4. Discussion

This project examined how eight weeks of WP versus CHO supplementation influenced physical performance and body composition in soldiers engaged in IET. IET is expected to increase all measures of physical performance [32]. However, these data suggest WP supplementation significantly improved push-up performance compared to CHO supplementation. These findings are supported by previous literature indicating that WP supplementation is beneficial for strength performance [20,21,22]. This finding is practically relevant with soldiers in the WP group performing on average 7 more push-ups relative to those in the CHO post-intervention after controlling for initial push-up performance. The push-up test is a requirement for graduation from IET, and 7 additional push-ups could enable an IET soldier to successfully graduate [4]. Improvements in push-up performance may benefit soldiers when performing tasks that mimic the pushing movement such as lifting/carrying equipment or pushing up to climb over walls. Therefore, these improvements in strength may also translate into improvements in performance in other areas of a soldier’s career beyond IET graduation. Conversely, lower push-up performance during IET doubles the risk of being discharged from training [33]. Additionally, IET soldiers who do not pass the APFT are required to receive additional training and possibly repeat the training program. This leads to higher training costs and delayed integration of IET soldiers into operational units for military service. Proper preparation is crucial as the pool of physically ready soldiers has been diminished by repeated deployments [34], musculoskeletal injury, and a small pool of available recruits resulting from poor health and fitness of the United States general population [35]. 

WP supplementation also significantly reduced FM compared to CHO supplementation. Specifically, the WP group lost an additional 1.8 kg of FM and had a larger effect size (Cohen’s d = 0.67) compared to CHO-supplemented soldiers (Cohen’s d = 0.40). Decreasing FM is an important and practical benefit to physical training when the intent is to optimize performance. Several studies have reported that WP supplementation promotes a reduction in FM in college-aged men over several months of resistance training compared to other protein supplements [36,37]. Moreover, rodent studies have suggested that WP mechanistically elicits lipolytic effects [38]. Conversely, a recent study in college-aged men (the same age group as IET soldiers) failed to demonstrate this fat loss effect when comparing WP to CHO and other supplementation groups (i.e., soy and leucine + CHO) over a 12-week period during resistance training [39]. However, IET differs from traditional resistance training given that it is comprised of strength as well as endurance training. Here, our results agree with the former studies [36,37] and suggest WP supplementation has a beneficial effect in reducing FM when administered during a strength and endurance conditioning training protocol.

There was no statistically significant benefit when comparing CHO versus WP supplementation on run performance. Specifically, there were large effect sizes for improvements in the metric regardless of supplement. Previous research suggests that additional intake of carbohydrate optimizes endurance performance [40]. The additional amino acids provided by WP may have also helped improve endurance performance by optimizing recovery. IET is physically demanding with participants consistently performing large volumes of training [8,9]. Studies in endurance athletes engaged in large volumes of training suggest WP improves recovery and reduced markers of muscle damage [23,24]. Thus, it is possible that WP helped maintain run performance relative to CHO supplementation by promoting recovery of IET soldiers, although this cannot be determined without a non-supplemented comparison group.

The lack of differences in FFM changes between groups was contrary to our hypothesis. It is notable that approximately 90% of the soldiers in the current study gained FFM. A previous study in IET soldiers reported only 36% of non-supplemented males gained FFM [10]. The authors reported that on average IET soldiers lost 1.4 kg of FFM and suggested that caloric deficits during training could have been the reason for the small percentage of IET soldiers who gained FFM. We have previously reported IET soldiers may exist in an estimated 595 calorie energy deficit during IET when no supplementation is provided [9]. In the current study, we supplied two WP or CHO servings per day, totaling approximately 580 additional daily calories during IET. Thus, in light of previous research, our findings may suggest that additional calorie intake itself may be beneficial for increasing FFM during IET. Future investigations should employ a non-supplemented control group to delineate responses to training in supplemented versus non-supplemented soldiers.

Self-reported diet logs indicated participants consumed a relatively high protein diet relative to body weight (1.5–1.7 g/kg) during the first week of training when no supplementation was provided. Protein intakes at these levels have been shown to be adequate for nitrogen balance and optimization of FFM response to resistance exercise training [11,41]. Thus, the high protein intake in both groups may have been adequate to support an optimal FFM response to IET training. The negligible between-group differences in FFM changes could also have been due to a variation in training experience and fitness levels prior to IET. A recent meta-analysis [42] revealed that protein supplementation is likely not as effective in untrained individuals compared to individuals who regularly participate in resistance training. The initial FFM in the soldiers in this study ranged from 47.0 to 80.0 kg across both groups, suggesting that there was a heterogenic distribution of muscle mass prior to training. Additionally, post hoc analysis indicated low initial FFM inversely correlated with change in FFM across training (*r* = −0.45, *p* < 0.001) indicating soldiers with low initial FFM experienced more robust increases in this variable with training. Hence, more research is needed in determining how baseline fitness and body composition levels affect the physiological responses to WP supplementation.

### Limitations

Certain limitations to this study exist. First, skinfolds were used to assess body composition, and this method has been typically viewed as a less reliable body composition test. Thus, while there was a high intraclass correlation between the raters herein, future investigations that examine the efficacy of WP supplementation in this population should use methodologies such as bioelectrical impedance or DEXA which have less variation in measures. It should also be noted that the fitness testing was collected by unit drill sergeants. As a result of multiple testers, interrater variability could have influenced our findings. However, drill sergeants are highly experienced and trained to administer these fitness assessments, and IET soldier graduation is based on the results of this fitness testing suggesting high fidelity of these data. The APFT is the Army fitness test of choice. Thus, we chose it as an assessment because of its applicability to the Army. Additionally, stringent training schedules during IET combined with large numbers of IET soldiers precluded the use of additional performance measures. This limited time availability also precluded the collection of certain biological variables (e.g., muscle biopsies or serial blood draws). Thus, future research in this regard is needed to determine the biochemical and physiological mechanisms by which WP or CHO supplementation may have influenced IET soldiers. CHO (versus other forms of protein) was chosen as a control group to determine if calorie-matched supplement intake from a non-protein source was beneficial for performance and body composition in IET where energy restriction may be a factor. Additionally, we sought to compare the influence of WP supplementation to CHO on run performance as previous literature has reported a benefit of added dietary CHO on aerobic performance. Another limitation is that participants were assigned to groups based on platoon and not randomized. This was done to prevent them from exchanging supplements with each other and to reduce errors during distribution of supplements. However, all platoons complete the same training, and all drill sergeants are under the same training guidance of unit leadership. Additionally, there were two different platoons in each supplement group to help reduce the influence of platoon training on the final results.

## 5. Conclusions

Twice daily supplementation of approximately 39 g of WP resulted in improved push-up performance and reduced FM compared to CHO supplementation. There was no statistical difference between supplement groups regarding changes in FFM, sit-up performance, or run performance. 

## Figures and Tables

**Table 1:** Summary of dietary intake during IET.

Group	Variable		Pre-NS	Post-NS	Post-SI
WP	Energy	(kcal/day)	2825 ± 611	2930 ± 681	3516 ± 681 *
		(kcal/kg/day)	40.3 ± 12.7	40.8 ± 11.1	49.0 ± 11.7 *
CHO		(kcal/day)	2624 ± 740	2766 ± 542	3348 ± 542 *
		(kcal/kg/day)	37.5 ± 13.7	38 ± 9.3	46.0 ± 9.9 *
WP	PRO	(g/day)	122 ± 25	124 ± 29	201 ± 29 *^,#^
		(g/kg/day)	1.7 ± 0.5	1.7 ± 0.4	2.8 ± 0.5 *^,#^
CHO		(g/day)	112 ± 32	113 ± 21	114 ± 21
		(g/kg/day)	1.6 ± 0.6	1.5 ± 0.4	1.6 ± 0.4
WP	CARB	(g/day)	371 ± 84	392 ± 96.6	430 ± 97 *
		(g/kg/day)	5.3 ± 1.7	5.5 ± 1.6	6.0 ± 1.6 *
CHO		(g/day)	349 ± 95	368 ± 85	495 ± 85 *^,#^
		(g/kg/day)	5.0 ± 1.7	5.0 ± 1.4	6.8 ± 1.5 *
WP	FAT	(g/day)	98 ± 27	100 ± 30.9	115 ± 31 *
		(g/kg/day)	1.4 ± 0.5	1.4 ± 0.5	1.6 ± 0.5 *
CHO		(g/day)	90 ± 31	98 ± 23.2	106 ± 23 *
		(g/kg/day)	1.3 ± 0.6	1.4 ± 0.4	1.5 ± 0.4 *

All data are food log data that are self-reported, averaged to one-day daily average intakes, and presented as means ± standard deviation values. Abbreviations: Pre-NS, pre-intervention when not supplementing; Post-NS, post-intervention values not including supplement; Post-SI, post-intervention values including supplement nutrition data; PRO, dietary protein; CARB, dietary carbohydrate; FAT, dietary fat; WP, whey protein group (*n* = 27); CHO, carbohydrate-placebo group (*n* = 28). Symbols: *, indicates Post > Pre (*p* < 0.05); #, indicates group differences at a given testing time point (*p* < 0.05).

**Table 2:** Summary of body composition during IET.

Variable	Group	Pre-Intervention	Post-Intervention
Body weight (kg)	WP	73.4 ± 12.7	73.2 ± 10.5
	CHO	72.3 ± 10.9	73.2 ± 7.9
FFM (kg)	WP	60.0 ± 7.9	64.2 ± 7.5
	CHO	60.1 ± 7.3	63.7 ± 6.1
Fat Mass (kg)	WP	13.5 ± 6.1	8.9 ± 4.2 *
	CHO	12.2 ± 6.1	9.5 ± 3.9 *

All data are presented as means ± standard deviation values. Body composition was assessed using 7-site skinfolds as described in the methods. Abbreviations: kg, kilograms; FFM, fat-free mass; WP, whey protein group (*n* = 34); CHO, carbohydrate-placebo group (*n* = 35); Symbols: *, indicates a significant group difference at post-training (*p* < 0.05).

**Table 3:** Summary of effect sizes.

Variable	Group	Mean Difference	95% CI	Units	Effect Size	Classification
FFM	WP	4.2	(3.1, 5.4)	kg	0.44	Medium
	CHO	3.6	(2.3, 4.9)	kg	0.42	Medium
FM	WP	−4.5	(−5.8, −3.2)	kg	−0.67	Large
	CHO	−2.7	(−4, −1.3)	kg	−0.4	Medium
PU	WP	6.8	(2.9, 10.7)	push-ups	0.41	Medium
	CHO	2.6	(−0.7, 6)	push-ups	0.18	Small
SU	WP	9.3	(5.4, 13.2)	sit-ups	0.62	Large
	CHO	8.9	(6.3, 11.5)	sit-ups	0.68	Large
Run	WP	−48.3	(−63, −33.6)	seconds	−0.56	Large
	CHO	−74.2	(−96.5, −51.9)	seconds	−0.74	Large

Mean differences along with 95% confidence intervals for the mean difference (lower limit, upper limit); Cohen’s d effect sizes are calculated for pre- to post-intervention changes in sit-up (SU), run time (Run), push-up performance (PU), fat-free mass (FFM) and fat mass (FM). Values in the positive direction indicate an improvement in the metrics for FFM, PU, SU, whereas negative values indicate an improvement in metrics for FM and Run (i.e., decreased FM indicates loss of FM and negative values for run performance indicate faster time at post vs. pre). Abbreviations: WP, whey protein group (*n* = 34); CHO, carbohydrate-placebo group (*n* = 35).

## Abbreviations

IET	Initial Entry Training
FM	Fat Mass
FFM	Fat Free Mass
WP	Whey Protein
CHO	Carbohydrate
RDA	Recommended Daily Allowance
USG	Urine Specific Gravity
ACSM	American College of Sports Medicine
APFT	Army Physical Fitness Test
